# Primary care patient experience and cancer screening uptake among women: an exploratory cross-sectional study in a Japanese population

**DOI:** 10.1186/s12930-017-0033-7

**Published:** 2017-02-07

**Authors:** Takuya Aoki, Machiko Inoue

**Affiliations:** 1 Department of Healthcare Epidemiology, School of Public Health in the Graduate School of Medicine, Kyoto University, Yoshida-Konoe-cho, Sakyo-ku, Kyoto, Kyoto Prefecture 606-8501 Japan; 2 Division of General Medicine and Primary Care, Beth Israel Deaconess Medical Center, Harvard Medical School, 330 Brookline Avenue, Boston, MA 02215 USA; 3 Department of Family and Community Medicine, Hamamatsu University School of Medicine, Shizuoka, 1-20-1 Handayama, Higashi-ku, Hamamatsu, Shizuoka Prefecture 431-3192 Japan

**Keywords:** Early detection of cancer, Patient experience, Primary health care, Process assessment (health care), Women’s health services

## Abstract

**Background:**

Patient experience and clinical quality, which are represented by preventive care measures such as cancer screening, are both widely used for the evaluation of primary care quality. The aim of this study was to examine the association between patient experience and cancer screening uptake among women in a Japanese population.

**Methods:**

We conducted a cross-sectional mail survey. The questionnaire was sent to 1000 adult female residents randomly selected from a basic resident register in Yugawara town, Kanagawa, Japan. We assessed patient experience of primary care using a Japanese version of Primary Care Assessment Tool (JPCAT) and uptake of breast and cervical cancer screening.

**Results:**

The overall response rate was 46.5%. Data were analyzed for 190 female participants aged 21–74 years who had a usual source of primary care. Multivariate logistic regression analyses revealed that the JPCAT total score was significantly associated with uptake of breast cancer screening [odds ratio (OR) per 1 standard deviation increase = 1.63; 95% CI 1.11–2.41], but not with uptake of cervical cancer screening (OR per 1 standard deviation increase = 1.47; 95% CI 0.97–2.24).

**Conclusions:**

Patient experience of primary care was associated with uptake of breast cancer screening among Japanese women. The results of our study might support the argument that patient experience of primary care and the clinical process of preventive care, such as breast cancer screening, are linked.

**Electronic supplementary material:**

The online version of this article (doi:10.1186/s12930-017-0033-7) contains supplementary material, which is available to authorized users.

## Background

In recent years, patient experience has attracted a lot of attention as one of the three pillars of quality health care, alongside clinical quality and patient safety [1]. Patient experience is the most effective measure of patient-centeredness, which is defined as providing care that is respectful of and responsive to patient preferences, needs, and values. This method enables objective assessment of quality of care by inquiring of patients about perceptions and events in the process of care, and has been increasingly used to assess the quality of primary care and hospital care in many countries [2]. According to previous studies, patient experience affects health outcomes through patient behavior such as adherence to treatment and healthcare resource use [3, 4].

It is no wonder that clinical quality is also a crucial aspect of healthcare quality. One of the important clinical processes in primary care is preventive care, such as cancer screening. However, in Japan, low cancer screening uptake rates have been recognized as a big issue, particularly in women. The breast and cervical cancer screening rates are still 43.4 and 42.1%, respectively, which are lower than the rates in other OECD countries [5–7], although incidences of the breast and cervical cancer have progressively increased. In Japan, the 2012 age-standardized incidence rates for the breast and cervical cancer were 64.3 and 24.0 per 100,000 women, respectively [8].

The Japanese unique primary care delivery system is one of the possible causes of the low cancer screening uptake among women. In Japan, primary care is typically delivered by specialists, who switch from hospital-based specialty practice to mixed primary/specialty care outpatient practice without emphasis on training for preventive care. Thus, generally, primary care providers, except for gynecologists, do not directly participate in breast and cervical cancer screening tests. However, there is no doubt that all primary care physicians should have a role in promoting cancer prevention activities for women in Japan as women have greater access to their primary care physician than any other physicians, and primary care physicians usually have a trusted and valued relationship with patients [9].

Previous findings from other countries revealed that patient experience correlates with the clinical process of care for prevention and disease management in primary care settings [10, 11]. Therefore, it is increasingly important to recognize the association between patient experience and other aspects of healthcare quality represented by clinical quality in order to improve our understanding of the quality of healthcare. However, it is unclear whether there is a similar association between patient experience and clinical quality in the Japanese setting. In this study, we specifically aimed to investigate the association between patient experience of primary care and uptake of breast and cervical cancer screening among Japanese women.

## Methods

We conducted a cross-sectional study to examine the association between patient experience of primary care and cancer screening uptake among women. Ethical approval was obtained from Hamamatsu University of author’s former affiliation (approval number E15-089).

### Subjects

Potential study participants were randomly selected from adult female residents in Yugawara Town in Kanagawa Prefecture, Japan, by using systematic random sampling from a basic resident register. Yugawara Town is located in the southern part of the Kanto area of eastern Japan. The total population of Yugawara Town was 26,442 according to the 2015 population census [12], with 35.8% of residents ≧65 years old. In terms of occupational structure, employment was provided by primary industry for 3.4%, secondary industry for 17.4%, and tertiary industry for 79.1%.

Of the potential participants, eligible participants were women aged 21–74 years who had at least one usual source of care (USC). We defined the eligible age range to cover the recommended age groups for both breast and cervical cancer screening (breast cancer screening: aged 50–74 years; cervical cancer screening: aged 21–65 years) by Japanese guidelines for breast and cervical cancer screening [13, 14], and recommendations from the United States Preventive Services Task Force (USPSTF) [15]. For this study, we used the same three questions and the algorithm in the JPCAT [16] as an original Primary Care Assessment Tool adult expanded version (PCAT-AE) [17] to identify an individual’s USC and the strength of that affiliation: (1) Is there a doctor that you usually go if you are sick or need advice about your health? (usual source); (2) Is there a doctor that knows you best as a person? (knows best); and (3) Is there a doctor that is most responsible for your health care? (most responsible). A participant was considered to have a USC if she answered positively to any one of the three questions.

We sent a self-administered questionnaire to total 1000 randomly selected adult women from the registers of sampling site. The data were collected between July and August 2015. Four weeks after the initial mailing, a reminder was sent out to increase the response rate. Regardless of whether the participants responded to the survey, they were given small gifts worth 200 JPY.

### Measures

#### Patient experience of primary care

We used the JPCAT [16] for data collection (Additional file 1). The JPCAT was based on the PCAT-AE [17], which was developed by Johns Hopkins Primary Care Policy Center, to measure the quality of primary care using patient experience in Japan. This 29-item tool comprises six multi-item subscales representing five primary care principles: first contact, longitudinally, coordination, comprehensiveness, and community orientation [18]. The JPCAT scoring system is structured as follows: each response on a five-point Likert scale is reduced by a factor of 1 and multiplied by 25. The score for each of the domains is computed as the mean value for all converted scale scores in that domain. Thus the domain scores range from 0 to 100 points, with higher scores indicating better performance. The total score is the mean of six domain scores and reflects an overall measure of the quality of core primary care principles. Previous work has shown that the JPCAT has good reliability and validity [16].

#### Uptake of cancer screening

The primary outcome measures in this study were uptake of breast and cervical cancer screening. Female participants answered questions about their breast and cervical cancer screening histories. According to Japanese guidelines for breast and cervical cancer screening [13, 14], and recommendations from the USPSTF [15], uptake of cancer screening was defined as completion of screening test within the last 2 years of the study period. Uptake of breast cancer screening was derived from the question “Have you completed breast cancer screening test within the last 2 years?” Similarly, uptake of cervical cancer screening was derived from the question “Have you completed cervical cancer screening test within the last 2 years?” Female participants answered the questions on binary scale (“yes” or “no”).

#### Covariates

Covariates were selected on the basis of a literature review to identify factors that may confound the association between patient experience and cancer screening uptake. We included covariates for age, years of education, household income, and self-rated health. All covariates, except for age, were evaluated as categorical variables by a self-administered questionnaire.

### Analyses

Descriptive statistics were obtained for the characteristics of the female respondents and the JPCAT scores. According to Japanese guidelines [13, 14] and recommendations from the USPSTF [15], responses from women aged 50–74 years were analyzed for breast cancer screening uptake, and responses from women aged 21–65 years were analyzed for cervical cancer screening uptake. We defined the eligible age ranges, which are the recommended age groups for screening by both Japanese and the USPSTF guidelines. Unadjusted association between the JPCAT total score and cancer screening uptake was analyzed by the Student’s *t* test. To determine whether the JPCAT total score was associated with cancer screening uptake, we used multivariate logistic regression analysis. The following possible confounders were included in the analysis: age, years of education, household income, and self-rated health. We had two primary outcomes; therefore, we used a Bonferroni correction to control type I error, and only those associations with *P* < 0.025 were considered to be significant. In addition, we performed exploratory analyses of the cancer screening uptake in relation to each domain score of the JPCAT. Missing data were uncommon for independent variables included in the regression model (0–5.1% missing data); therefore, we performed complete case analyses.

According to the sample size formula shown in a previous study, events per variable values of ≥10 were necessary for logistic regression analysis [19]. We estimated a minimum sample size of 125 because the maximum number of variables was five in this study, and the proportion of cancer screening uptake within the last 2 years in our target population was assessed to be 0.40 according to the 2013 Japanese comprehensive survey of living conditions [7]. We used SPSS version 23 for statistical analyses.

## Results

**Fig. 1:**
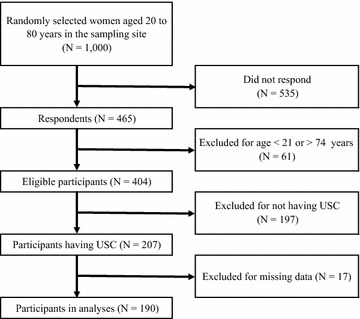
Participant flow chart. *USC*  usual source of care

A total of 465 (46.5%) individuals responded to the mail survey. In the responses, we excluded participants aged <21 or >74 years, and participants who did not have a USC. We performed analyses of the 190 participants with complete data for the variables (Fig. 1).

Table 1 shows the individual characteristics of the 190 eligible participants surveyed. Among the participants, 47.9% were found to be aged ≥65, and 55.8% of participants had no college education. The proportion of participants who underwent breast and cervical cancer screening within 2 years were both 43.2%.

**Table 1:** Participants’ characteristics (N = 190)

Characteristic	Number (%)
Age (years)	
21–49	35 (18.4)
50–64	64 (33.7)
65–74	91 (47.9)
Education	
Less than high school	25 (13.2)
High school	81 (42.6)
Junior college	63 (33.2)
More than or equal to college	21 (11.1)
Annual household income (million JPY)	
<2.00 (≒18,000 US dollar)	49 (25.8)
2.00–4.99	100 (52.6)
≧5.00	41 (21.6)
Self-rated health	
Very good	25 (13.2)
Good	45 (23.7)
Neutral	66 (34.7)
Poor	44 (23.2)
Very poor	10 (5.3)
Breast cancer screening	
Uptake within 2 years	82 (43.2)
Cervical cancer screening	
Uptake within 2 years	82 (43.2)

Table 2 shows the mean and standard deviation of the JPCAT scores. The average JPCAT total score was 52.3 out of 100 points; the most highly scored domain was longitudinally (65.7), and the most poorly scored domain was comprehensiveness (services provided) (37.2). The univariate associations between patient experience of primary care and outcomes are also shown in Table 2. The JPCAT total score was significantly associated with uptake of breast cancer screening (*P* = 0.008), and community orientation domain score was associated with uptake of breast cancer screening. However, the association between the JPCAT total score and uptake of cervical cancer screening was not statistically significant.

**Table 2:** Distribution^a^ of JPCAT, and unadjusted correlation with breast and cervical cancer screening uptake

Scale	Total (N = 190)	Breast cancer screening^b^ (N = 155)		*P* value^d^	Cervical cancer screening^c^ (N = 111)		*P* value^d^
		Uptake (N = 64)	Non-uptake (N = 91)		Uptake (N = 61)	Non-uptake (N = 50)	
JPCAT							
Total score	52.3 (15.6)	58.4 (15.7)	51.9 (14.1)	0.008	53.7 (16.8)	49.9 (14.6)	0.238
First contact	46.3 (25.5)	52.9 (22.9)	50.4 (24.0)	0.514	43.9 (26.8)	43.3 (25.1)	0.912
Longitudinality	65.7 (18.4)	70.0 (17.1)	65.0 (19.1)	0.096	65.6 (18.2)	64.6 (20.0)	0.782
Coordination	57.7 (25.3)	64.8 (24.3)	58.1 (24.2)	0.096	58.2 (29.7)	51.7 (21.2)	0.196
Comprehensiveness (services available)	56.9 (23.7)	60.2 (22.4)	54.5 (23.6)	0.132	59.9 (25.0)	55.4 (25.3)	0.349
Comprehensiveness (services provided)	37.2 (26.7)	43.3 (28.4)	35.0 (24.3)	0.055	44.1 (29.2)	36.6 (26.3)	0.167
Community orientation	49.6 (21.2)	58.3 (21.1)	48.4 (19.1)	0.003	49.2 (22.5)	46.9 (21.8)	0.586

*JPCAT* Japanese version of Primary Care Assessment Tool

^a^Mean (SD)

^b^Used responses from women aged 50–74 years

^c^Used responses from women aged 21–65 years

^d^ *P* value by t test

Table 3 shows the results of the multivariate logistic regression analyses modeling the association between patient experience of primary care and uptake of breast cancer screening. We used responses from women aged 50–74 years for the analyses. After adjustment for possible confounders, the JPCAT total score was positively associated with uptake of breast cancer screening [odds ratio (OR) per 1 standard deviation increase = 1.63; 95% CI 1.11–2.41]. Community orientation had the strongest association with uptake of breast cancer screening (OR per 1 standard deviation increase = 1.80; 95% CI 1.18–2.70), followed by coordination (OR per 1 standard deviation increase = 1.57; 95% CI 1.08–2.27).

**Table 3:** Factors associated with breast cancer screening uptake^a^ (N = 155)

Scale	aOR (95% CI)^b^	*P* value
JPCAT		
Total score	1.63 (1.11–2.41)	0.013
First contact	1.14 (0.77–1.67)	0.486
Longitudinality	1.29 (0.90–1.85)	0.173
Coordination	1.57 (1.08–2.27)	0.020
Comprehensiveness (services available)	1.33 (0.91–1.88)	0.141
Comprehensiveness (services provided)	1.24 (0.87–1.79)	0.232
Community orientation	1.80 (1.18–2.70)	0.006

*JPCAT* Japanese version of Primary Care Assessment Tool, *aOR* adjusted odds ratio

^a^Used responses from women aged 50–74 years. Adjusted for age, years of education, annual household income, and self-rated health

^b^Per 1 standard deviation increase

Table 4 shows the results of the multivariate logistic regression analyses modeling the association between patient experience of primary care and uptake of cervical cancer screening. We used responses from women aged 21–65 years for the analyses. In contrast with the results of breast cancer screening, the JPCAT total score was not significantly associated with uptake of cervical cancer screening (OR per 1 standard deviation increase = 1.47; 95% CI 0.97–2.24). None of the domains of the JPCAT except for coordination were significantly associated with uptake of cervical cancer screening.

**Table 4:** Factors associated with cervical cancer screening uptake^a^ (N = 111)

Scale	aOR (95% CI)^b^	*P* value
JPCAT		
Total score	1.47 (0.97–2.24)	0.068
First contact	1.19 (0.75–1.88)	0.435
Longitudinality	1.12 (0.76–1.63)	0.587
Coordination	1.53 (1.00–2.33)	0.048
Comprehensiveness (services available)	1.13 (0.77–1.64)	0.533
Comprehensiveness (services provided)	1.41 (0.95–2.09)	0.090
Community orientation	1.31 (0.86–1.99)	0.218

*JPCAT* Japanese version of Primary Care Assessment Tool, *aOR* adjusted odds ratio

^a^Used responses from women aged 21–65 years. Adjusted for age, years of education, annual household income, and self-rated health

^b^Per 1 standard deviation increase

## Discussion

Our results revealed that patient experience of primary care was associated with uptake of breast cancer screening among Japanese women. This association persisted after adjustment for possible confounders. In addition, associations with breast cancer screening uptake were statistically significant for the coordination domain score and community orientation domain score. By contrast, patient experience of primary care was not significantly associated with uptake of cervical cancer screening, although there was a trend for positive association between them.

Previous studies from other countries showed that patient experience was associated with clinical quality including cancer screening in primary care [10, 20, 21]. However, these findings were from settings in which primary care providers directly participate in breast and cervical cancer screening tests, unlike Japan. The results of our study could act as an extension of the research findings on the association between patient experience and clinical process of preventive care. In addition, the coordination domain of the JPCAT was associated with breast cancer screening uptake in this study. This finding suggests that better coordination between primary care providers and specialists including gynecologists may be effective in encouraging breast cancer screening in Japan, reflecting a unique primary care delivery system.

Community-oriented primary care (COPC) is a continuous process by which primary care is provided to a defined community on the basis of its assessed health needs through the planned integration of public health practice with the delivery of primary care services. This link with public health places health promotion and disease prevention at the forefront of the COPC concept [22]. In concordance with this concept of COPC, we found that the community orientation domain of the JPCAT was significantly associated with uptake of breast cancer screening.

In contrast to the results of breast cancer screening, the association between patient experience and cervical cancer screening was not statistically significant. These results might be caused by the difference in sample size, and also indicate the possibility that primary care providers in Japan have not been able to sufficiently demonstrate their role in younger residents’ preventive care activities, in contrast to the case with older residents. In concordance with this finding, previous study showed that many Japan’s primary care physicians have limited training in preventive care for young women and few provide it [23]. However, the potential association between contribution of primary care to residents’ preventive care activities and age group requires further study.

This is the first study revealing the association between patient experience and clinical quality represented by the process of preventive care in Japan. Characteristics of the Japanese primary care delivery system are helpful to reinforce the findings of previous studies about the association between these two domains. The PCAT is an established measure for the evaluation of patient experience of primary care internationally, and represents the core principles of primary care.

Our study had several potential limitations. First, there was a concern about the low response rate. However, in the case of patient experience surveys, there is little evidence that a low response rate introduces selective non-response bias [24]. Second, although self-reported survey is a useful method for evaluating uptake of cancer screening in population-based studies [25], recall bias may be a factor in this type of survey. For example, participants who have good patient experience possibly over-report preventive care activities. Social desirability bias might also limit the study if such participants responded more favorably than others and overreported uptake of cancer screening. Third, we could not assess unnecessary cancer screening according to the guidelines as a marker of lower clinical quality in this study. Fourth, the data were cross-sectional and a causal relationship between patient experience of primary care and uptake of cancer screening cannot be definitely established. Fifth, this study population covered only restricted rural area in Japan, thus our study may have limited external validity.

## Conclusions

We found that patient experience of primary care was associated with uptake of breast cancer screening among women in the Japanese setting. The results of our study might support the argument that patient experience of primary care and the clinical process of preventive care, such as breast cancer screening, are linked.

## Additional file

**Additional file 1.** Item contents of the Japanese version of Primary Care Assessment Tool.

## Abbreviations

JPCAT: Japanese version of Primary Care Assessment Tool
USC: usual source of care
PCAT-AE: Primary Care Assessment Tool adult expanded version
USPSTF: United States Preventive Services Task Force
OR: odds ratio
COPC: community-oriented primary care
