# Supplementary material for: Primary care patient experience and cancer screening uptake among women: an exploratory cross-sectional study in a Japanese population
Source: Asia Pac Fam Med. 2017 Feb 7;16:3. doi: 10.1186/s12930-017-0033-7 (PMC5297167; doi:10.1186/s12930-017-0033-7)
Supplement: Supplementary file 1 — Additional file 1. Item contents of the Japanese version of Primary Care Assessment Tool. [file 12930_2017_33_MOESM1_ESM.pdf]

Additional file 1. Item contents of the Japanese version of Primary Care Assessment Tool

Domain	Item content
First contact	When your Primary Care Practice is closed, is there a phone number you can call when you get sick?
	When your Primary Care Practice is closed on Saturday and Sunday and you get sick, would someone from there see you the same day?
	When your Primary Care Practice is closed and you get sick during the night, would someone from there see you that night?
Longitudinality	Does your Primary Care Physician (PCP) give you enough time to talk about your worries or problems?
	Do you feel comfortable telling your PCP about your worries or problems?
	Does your PCP know you very well as a person, rather than as someone with a medical problem?
	Does your PCP know what problems are most important to you?
	Does your PCP know your complete medical history?
Coordination	Have you ever had a visit to any kind of specialist or special service?
	Did your PCP suggest you go to the specialist or special service?
	Did your PCP discuss with you different places you could have gone to get help with that problem?
	Did your PCP or someone working with your PCP help you make the appointment for that visit?
	Did your PCP write down any information for the specialist about the reason for the visit?
	Does your PCP know what the results of the visit were?
Comprehensiveness	For each one, please indicate whether it is available at your PCP's office.
(services available)	Counseling for mental health problems
	Changes in mental or physical abilities that are normal with getting older
	Counseling related to dementia
	Counseling related to abuse
	Counseling related to personal preferences about end of life issues
Comprehensiveness	In visits to your PCP, are any of the following subjects discussed with you?
(services provided)	Advice about appropriate exercise for you
	Advice about regular bowel movements
	Advice about over-the-counter medications
	Advice about medical information in the media: on TV, in the newspaper, etc.

	Advice about a healthy balance of work and rest
Community orientation	Does anyone at your PCP's office ever make home visits?
	Does your PCP know about the important health problems of your community?
	Does your PCP get opinions and ideas from people that will help to provide better health care?
	Does your PCP investigate whether the available health care is meeting the needs of the community?
	Does your PCP investigate the concerns people have about health problems in your community?

Aoki T, Inoue M, Nakayama T. Development and validation of the Japanese version of Primary Care Assessment Tool. Fam Pract. 2016;33:112–7.
